# Supplementary material for: MetaQTL: a package of new computational methods for the meta-analysis of QTL mapping experiments
Source: BMC Bioinformatics. 2007 Feb 8;8:49. doi: 10.1186/1471-2105-8-49 (PMC1808479; doi:10.1186/1471-2105-8-49)
Supplement: Additional File 7 — MetaQTL Package : jar file and tutorial. This Zip archive contains both the MetaQTL JAR file and the files of the tutorial. [file 1471-2105-8-49-S7.zip › org.inra.metaqtl/doc/org/metaqtl/ChromCluster.ClusterGraph.html]

ChromCluster.ClusterGraph


|  |  |  |
| --- | --- | --- |
| **Overview** \| **Package** \| **Class** \| **Use** \| **Tree** \| **Deprecated** \| **Index** \| **Help** | |  |
| **PREV CLASS**   **NEXT CLASS** | **FRAMES**    **NO FRAMES**     **All Classes** |  |
| SUMMARY: NESTED \| FIELD \| CONSTR \| METHOD | DETAIL: FIELD \| CONSTR \| METHOD |  |


---


## org.metaqtl Class ChromCluster.ClusterGraph

```
java.lang.Object
  org.metaqtl.ChromCluster.ClusterGraph
```

**Enclosing class:**: ChromCluster

---

``` public class ChromCluster.ClusterGraph extends java.lang.Object ```

---

| **Field Summary** | |
| --- | --- |
| `java.util.Hashtable` | `adjTable` |
| `ChromCluster` | `cluster` |


**Constructor Summary**

`ChromCluster.ClusterGraph(ChromCluster cluster)`


| **Method Summary** | |
| --- | --- |
| `void` | `addEdge(Chromosome cur1, Chromosome cur2, CMarkerSequence cms)` |
| `java.util.Hashtable` | `getAdjacentTable()` |
| `java.lang.String[]` | `getVLabels()` |
| `boolean` | `isConnected(int nMrk)`             Test if the graph is connected |
| `void` | `summarize(java.io.OutputStream stream)` |

**Methods inherited from class java.lang.Object**

`clone, equals, finalize, getClass, hashCode, notify, notifyAll, toString, wait, wait, wait`

**Field Detail**

### adjTable

```
public java.util.Hashtable adjTable
```

---


### cluster

```
public ChromCluster cluster
```


**Constructor Detail**

### ChromCluster.ClusterGraph

```
public ChromCluster.ClusterGraph(ChromCluster cluster)
```

**Parameters:**: `cluster` -


**Method Detail**

### isConnected

```
public boolean isConnected(int nMrk)
```

:   Test if the graph is connected

    :   **Returns:**

---


### addEdge

```
public void addEdge(Chromosome cur1,
                    Chromosome cur2,
                    CMarkerSequence cms)
```

:   **Parameters:**: `cur1` -: `cur2` -: `nmc` -

---


### getAdjacentTable

```
public java.util.Hashtable getAdjacentTable()
```

:   **Returns:**

---


### summarize

```
public void summarize(java.io.OutputStream stream)
```

---


### getVLabels

```
public java.lang.String[] getVLabels()
```


---


|  |  |  |
| --- | --- | --- |
| **Overview** \| **Package** \| **Class** \| **Use** \| **Tree** \| **Deprecated** \| **Index** \| **Help** | |  |
| **PREV CLASS**   **NEXT CLASS** | **FRAMES**    **NO FRAMES**     **All Classes** |  |
| SUMMARY: NESTED \| FIELD \| CONSTR \| METHOD | DETAIL: FIELD \| CONSTR \| METHOD |  |


---
